# Supplementary figures and images for: Splice variants of DOMINO control Drosophila circadian behavior and pacemaker neuron maintenance
Source: PLoS Genet. 2019 Oct 28;15(10):e1008474. doi: 10.1371/journal.pgen.1008474 (PMC6837581; doi:10.1371/journal.pgen.1008474)

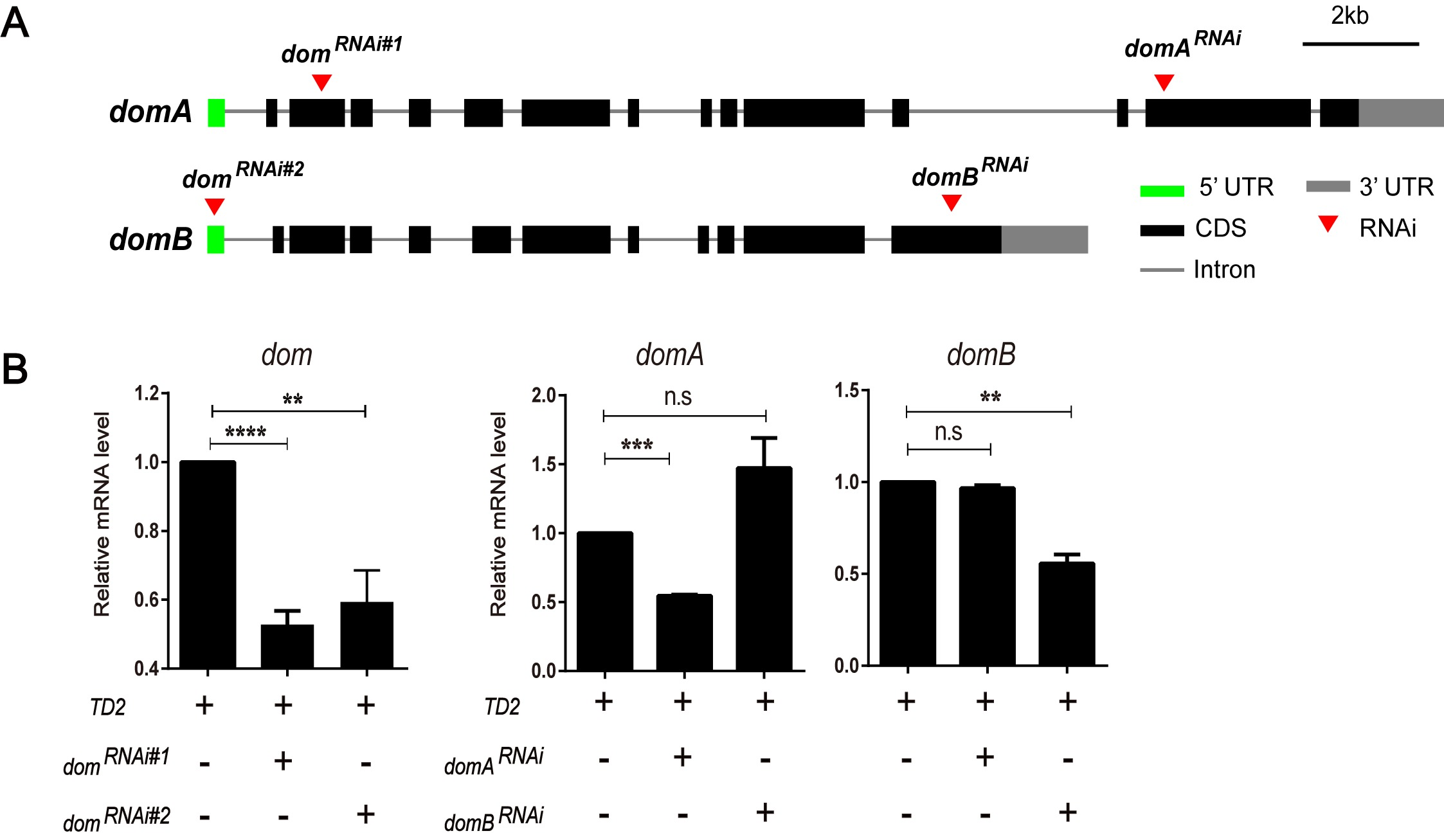

Supplement: S1 Fig — A. Schematic map of the domA and domB locus. Regions of the domA and domB mRNA targeted by the four specific RNAi lines. domRNAi#1 and domRNAi#2 are RNAi lines targeting different regions of dom. domARNAi and domBRNAi are short hairpin RNAi lines specifically targeting domA or domB isoform CDS regions. B. Quantitative RT-PCR showing the expression of dom, domA and domB. Flies were collected at ZT1. Downregulation of dom (domRNAi#1 and domRNAi2) in circadian neurons decreased dom mRNA levels, comparing to the controls. Downregulation of domA and domB (domARNAi and domBRNAi) in circadian neurons specifically decreased domA and domB mRNA levels, comparing to the relative controls. Error bars correspond to SEM. n.s., nonsignificant; **p < 0.01;***p < 0.001;****p < 0.0001 as determined by t-test. (TIF) [file pgen.1008474.s001.tif]

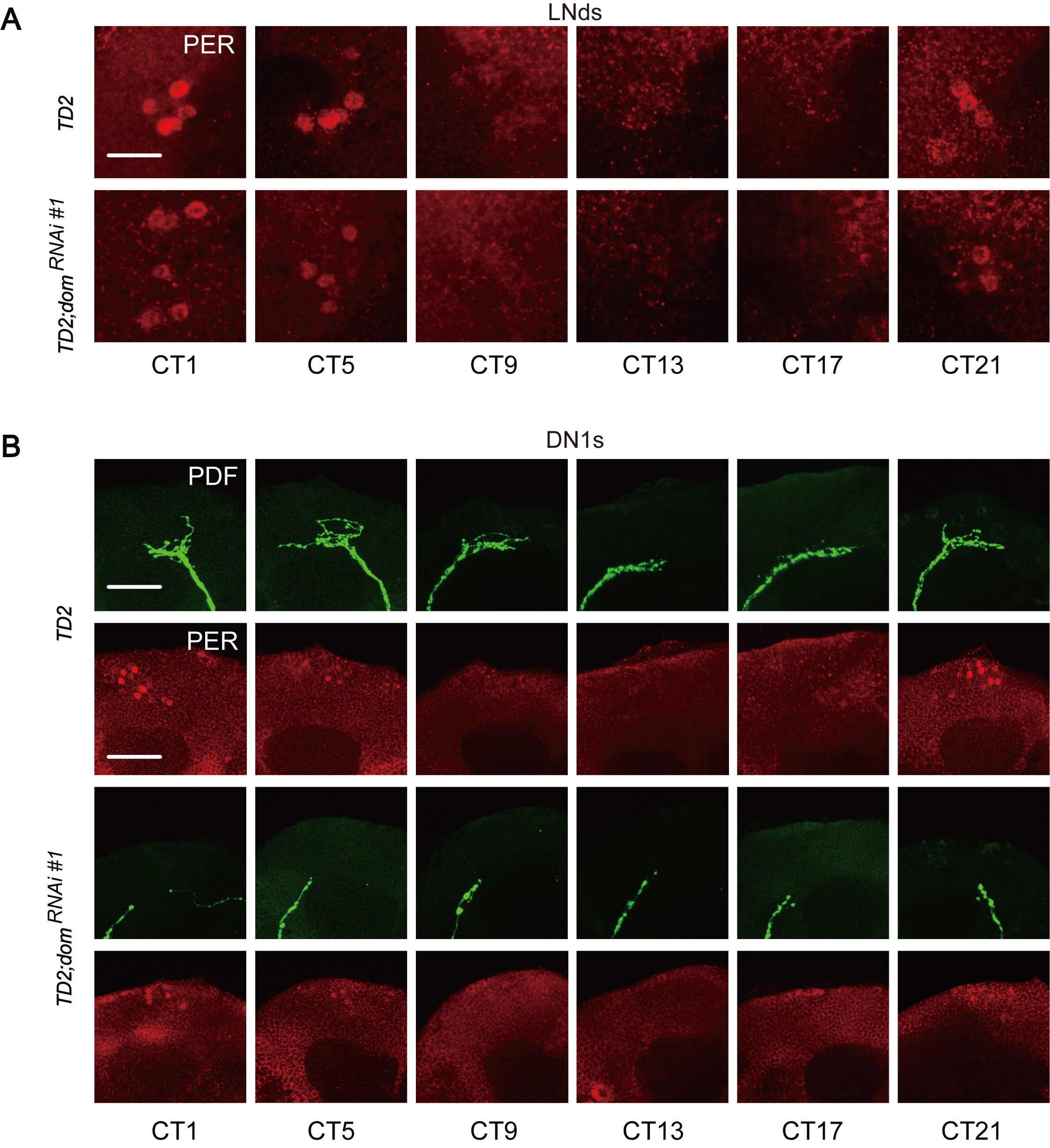

Supplement: S2 Fig — A-B. Whole mount immunostaining showing the expression pattern of PER in LNds and DN1s. Red is PER and green is PDF. Flies were entrained for 4 days in LD and transferred to DD and dissected every 4 h on the fifth day. Downregulation of DOM decreased PER levels at CT1-9 and CT17 in LNds, while reduced PER levels at CT1-5 and CT17-21 in DN1s (Scale bar: LNds, 50 um; DN1s, 150 um). (TIF) [file pgen.1008474.s002.tif]

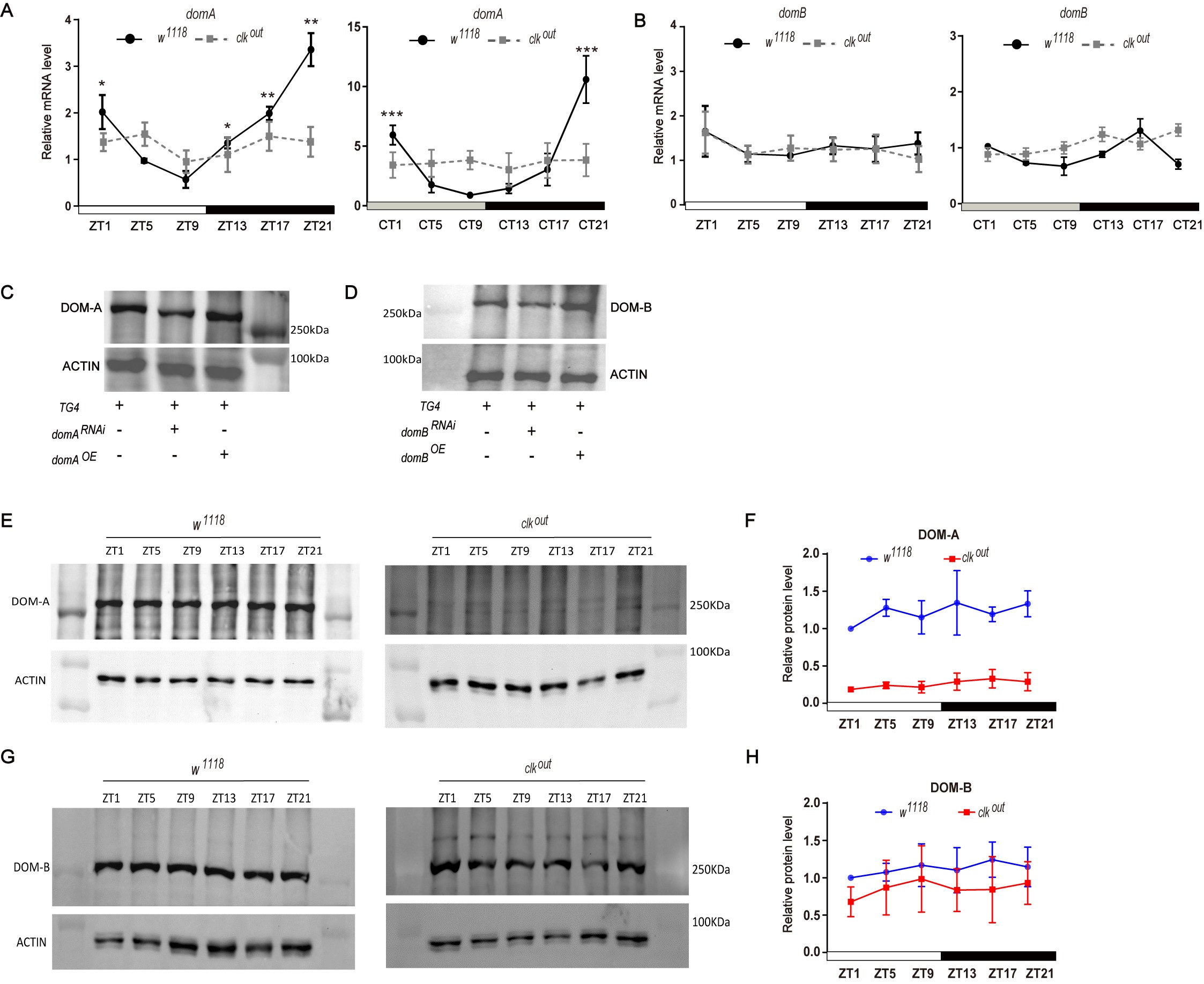

Supplement: S3 Fig — A-B. Quantitative RT-PCR showing the expression patterns of domA and domB in w1118 and Clkout flies heads. Flies were collected at the indicated time points (ZT = Zeitgeber Time or CT = circadian time). Dom-A exhibited a strong oscillation pattern with a trough around ZT9 and CT9 while has a peak expression near ZT21 and CT21 in w1118 flies heads, while the oscillation of dom-A expression was abolished in Clkout. There is no obvious oscillation for dom-B expression in w1118 and Clkout flies heads both ZT and CT conditions. C-D. Validation of specificity for domA and domB shRNA knockdowns and overexpression. UAS-domA, UAS-domB and UAS-shRNA of domA and domB males for domA and domB were crossed with tim-Gal4 females. F1 offspring adult brains are probed with anti-DOM-A and anti-DOM-B antibodies in western blot. ACTIN signals provided controls. E-H. Western blot showing the expression patterns of DOM-A and DOM-B in w1118 and Clkout flies heads. Flies were collected at the indicated time points (ZT = Zeitgeber Time). DOM-A did not show a strong oscillation pattern in w1118 flies heads, while the expression levels of DOM-A were remarkably decreased in Clkout. There is no obvious change for DOM-B expression in w1118 and Clkout flies heads. Band intensity was calculated and analyzed with the Image J. Error bars correspond to SEM. *P < 0.05;**p < 0.01,***p < 0.001 as determined by the t-test. (TIF) [file pgen.1008474.s003.tif]

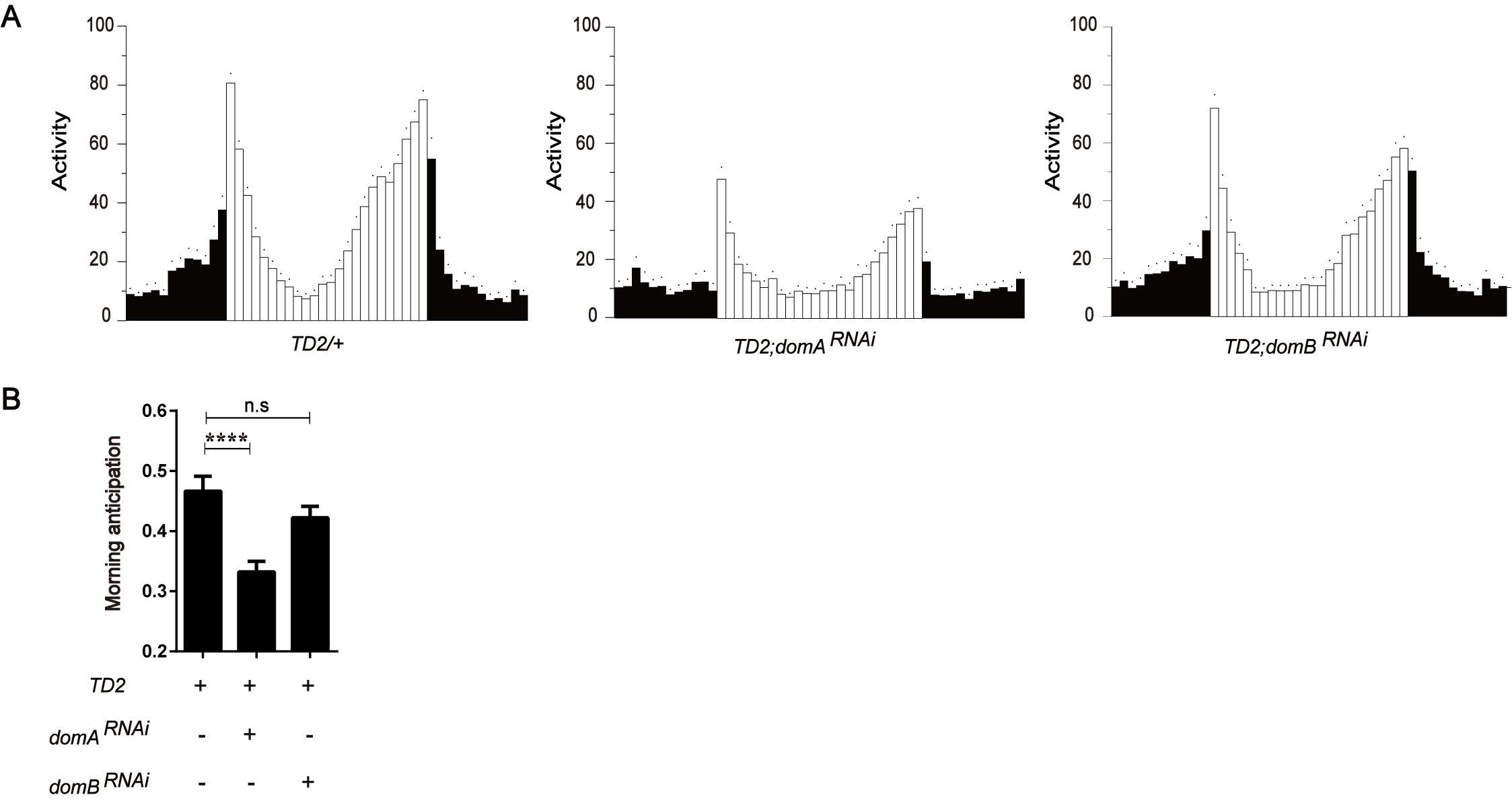

Supplement: S4 Fig — A. Average locomotor activity of flies of different genotypes under 3 days of 12:12 hr LD conditions. Dark activity bars represent the night, and white bars represent the day. Comparing to the control (left panel),morning anticipation was severely disrupted in domA shRNAi lines (middle panel),while morning anticipation was normal in domB downregulation flies (Right panel). B. Morning anticipation was calculated following the method described before. Error bars correspond to SEM. n.s., nonsignificant;****p < 0.0001 as determined by t-test. (TIF) [file pgen.1008474.s004.tif]

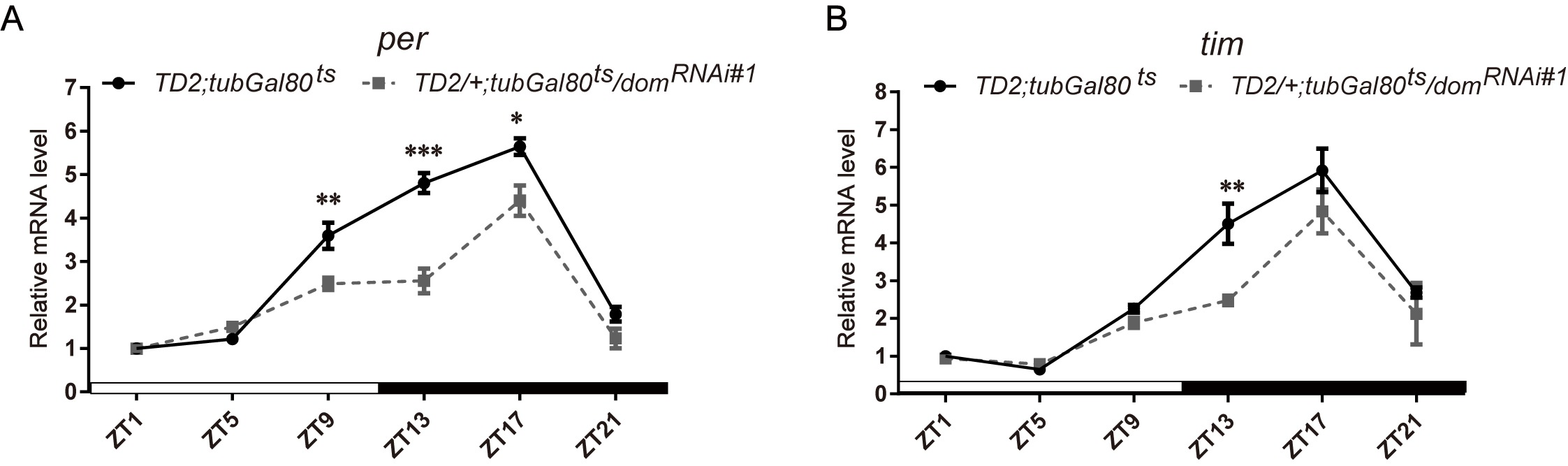

Supplement: S5 Fig — A-B. Quantitative RT-PCR showing the expression of per and tim. Flies were collected at the indicated time points (ZT = Zeitgeber Time). Downregulation of dom in adult stage decreased per and tim mRNA levels. (TIF) [file pgen.1008474.s005.tif]

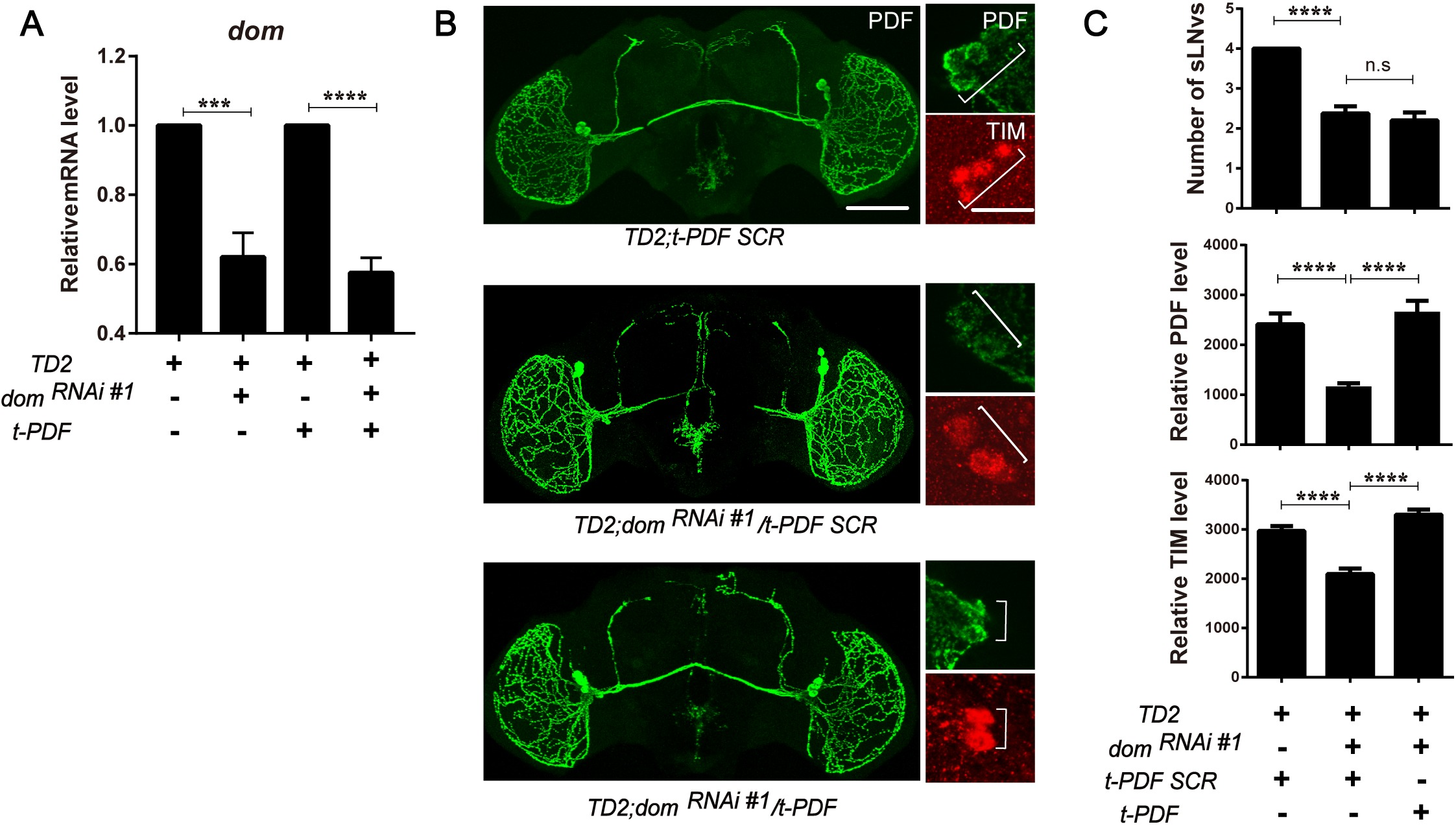

Supplement: S6 Fig — A. Quantitative RT-PCR showing the expression of dom. Flies were collected at ZT1. Downregulation of dom (domRNAi#1) in circadian neurons decreased dom mRNA levels (positive control). Dom mRNA level is still reduced by dom RNAi even expressing the membrane-tethered PDF. Error bars correspond to SEM. ***p < 0.001;****p < 0.0001 as determined by t-test. B. Representative confocal images of brains of dom RNAi flies expressing the membrane-tethered PDF or scrambled PDF. Flies were entrained for 4 days in LD 25°C, and brains were dissected at ZT23 for anti-PDF antibody (green) and anti-TIM antibody (red). From top to bottom: (Top panel) fly brain expressing the membrane-tethered scrambled PDF; (middle panel) domRNAi#1 flies expressing a membrane-tethered scrambled PDF; and (bottom panel) domRNAi#1 flies expressing the membrane-tethered PDF. Confocal images are whole brain and soma of sLNvs from left to the right (Scale bar: whole brain, 500 um; sLNvs, 50 um). C. Quantification of the number and relative PDF and TIM levels of sLNvs. For each genotype, totally, 14–20 flies brains and 30–55 neurons were used for quantification of the staining. Error bars correspond to SEM. n.s., nonsignificant;***p < 0.001, ****p < 0.0001 as determined by t-test. (TIF) [file pgen.1008474.s006.tif]
