# Supplementary material for: Splice variants of DOMINO control Drosophila circadian behavior and pacemaker neuron maintenance
Source: PLoS Genet. 2019 Oct 28;15(10):e1008474. doi: 10.1371/journal.pgen.1008474 (PMC6837581; doi:10.1371/journal.pgen.1008474)
Supplement: S1 Table — (PDF) [file pgen.1008474.s007.pdf]

Flies were raised at 25 °C and tested behavior at 25°C[a]				
**Genotype**	**N[b]**	**Rhythmic (%)[c]**	**Period (hr) ± S.D.**	**Power ± S.D.[d]**
tim-GAL4,UAS-dicer2(=TD2)/+	71	95.8	24.3±0.07	88.48±22.6
pdf-GAL4,UAS-dicer2 (=PD2)/+	56	96.4	24.3±0.09	95.9±25.2
TD2/ +; pdf-G80/+	72	87.5	24.8±0.2	75.9±12.6
domRNAi #1 [e]/+	54	85.2	24.2±0.1	91.7±5.7
domRNAi #2 [f] /+	55	98.2	24.3±0.06	107.9±4.7
sh-domA [g]/+	56	85.7	24.2±0.07	102.3±5.4
sh-domB [h]/+	40	90	24.2±0.1	98.8±5.8
TD2/+; luc RNAi /+	16	100	23.6±0.1	130.6±10.1
PD2/+; luc RNAi /+	16	93.8	24.2±0.1	10.6.3±8.4
TD2/+; pdf-G80/luc RNAi	16	93.8	23.7±0.1	98.8±5.8
TD2/+; dom RNAi #1/+	62	30.6	25.7±0.5	46.5±11.6
TD2/+; dom RNAi #2 /+	87	41.4	25.3±0.7	41.3±10.4
TD2/+; sh-domA /+	54	20.4	26.8±1.3	32.1±7.1
TD2/+; sh-domB /+	97	91.8	26.1±0.1	98.6±18.9
PD2/+; dom RNAi #1/+	52	34.6	24.8±1.3	53.1±8.0
PD2/+; dom RNAi #2 /+	71	67.6	26.3±0.5	62.1±18.1
PD2/+; sh-domA /+	76	32.9	24.4±0.7	43.2±17.2
PD2/+; sh-domB /+	71	97.2	26.3±0.8	95.3±12.5
TD2/+; pdf-G80/dom RNAi #1	46	89.1	25.0±0.1	81.5±9.7
TD2/+; pdf-G80/dom RNAi #2	88	73.9	25.2±0.2	76.3±12.6
TD2/+; pdf-G80/ sh-domA	31	29	25.3±0.7	37.9±5.1
TD2/+; pdf-G80/ sh-domB	69	89.9	25.7±0.1	104.2±11.1
W1118;TD2/+;sh-domA	117	23.1	26.1±0.2	37.6±3.4
W1118;TD2/+;sh-domB	111	75.7	25.6±0.2	81.0±3.6

domB oe/+	82	70.7	24.0±0.06	83.2±4.4
TD2/domB oe;sh-domA /+	38	13.2	24.7±0.7	46.8±5.7
TD2/domB oe;sh-domB /+	96	82.3	24.6±0.07	74.0±3.3
domA oe/+	83	95.2	23.7±0.04	106.5±3.8
TD2/+;/domA oe/sh-domA	78	70.5	24.3±0.1	67.3±4.4
TD2/+;/domA oe/sh-domB	94	71.3	25.9±0.09	73.8±3.5
TD2/+;P1B3/ +	39	82.1	24.0±0.2	92.1±4.7
TD2/+; P1B3/domRNAi [# 1]	64	26.6	25.5±0.3	46.3±6.5
TD2/+; P1B3/domRNAi [# 2]	61	54.1	25.5±0.3	57.0±5.2
TD2/+; P1B3/sh-domA	63	27.0	25.5±0.5	48.3±1.6
TD2/+; P1B3/sh-domB	47	72.3	25.6±0.2	76.4±6.8
TD2/+;t-PDF 58F1A/+	60	66.7	24.1±0.3	72.5±19.9
TD2/+;t-PDF 58F1A/domRNAi [# 1]	53	64.2	24.0±0.6	72.0±18.5
TD2/+; t-PDF 58F1A/domRNAi [# 2]	67	89.6	24.3±0.5	88.2±15.3
TD2/+; t-PDF 58F1A/sh-domA	74	71.6	24.2±0.3	74.1±7.0
TD2/+; t-PDF 58F1A/sh-domB	30	83.3	24.2±0.3	106.7±40
**Flies were raised at 18 °C and tested behavior at 30°C**[a]				
TD2/+;TUBgal80TS/+	117	100	23.9±0.3	103.8±9.8
TD2/+; TUBgal80TS /dom RNAi [# 1]	164	95.7	24.9±0.3	77.6±3.8
TD2/+;TUBgal80TS/dom RNAi [# 2]	85	91.8	25.4±0.2	90.7±25.4
TD2/+;TUBgal80TS/sh-domA	90	88.8	26.2±0.1	74.8±7.2
TD2/+;TUBgal80TS/sh-domB	88	90.9	25.0±0.4	111.8±28.4
**Flies were raised at 30°C and tested behavior at 18°C**[a]				
TD2/+;TUBgal80TS/+	42	69.1	24.3±0.2	53.0±4.8
TD2/+; TUBgal80TS /dom RNAi [# 1]	61	26.2	24.1±0.5	36.7±3.8
TD2/+;TUBgal80TS/dom RNAi [# 2]	41	9.8	24.3±0.6	28.8±2.8
TD2/+;TUBgal80TS/sh-domA	0			

TD2/+;TUBgal80TS/sh-domB	70	52.9	24.9±0.1	49.8±4.8

[a]: Flies were raised or tested behavior at indicated temperatures (18° C, 25° C, and 30° C) for behavior flies were

exposed to 4 d of 12:12 LD followed by 6 d of DD.

[b]: Total number of flies that survived until the end of the testing period.

[c]: Percentage of flies having a power value ≥20.

[d]: Relative measure of the strength or amplitude of the rhythm.

[e]: *dom RNAi*[#1]; Bloomington number 31054.

[f]: *dom RNAi*[#2]; Bloomington number 38941.

[g]: *sh-domA*; from Dr. Peter B. Becker.

[h]: *sh-domB;* from Dr. Peter B. Becker.
