# Supplementary material for: Splice variants of DOMINO control Drosophila circadian behavior and pacemaker neuron maintenance
Source: PLoS Genet. 2019 Oct 28;15(10):e1008474. doi: 10.1371/journal.pgen.1008474 (PMC6837581; doi:10.1371/journal.pgen.1008474)
Supplement: S2 Table — (PDF) [file pgen.1008474.s008.pdf]

Table S2. Primers used in this study.

**Primers**	**Sequence(5'-3')**	**Experiment**
per fwd	TGACCGAATCCCTGCTCAAT	*period* qPCR
per rev	CTTTTTATCCCGTGGCCTGG	*period* qPCR
tim fwd	CACTTCCGCAACAACAGAGT	*timless* qPCR
tim rev	ACTCCGCAGGGTCAGTTTAA	*timless* qPCR
clk fwd	GCAGGAAATCGCGTAATCTCA	*clock* qPCR
clk rev	ATCGGTGGCCTCATTATGATTTT	*clock* qPCR
dom fwd	GGAACAGGATGAACAGGAGGAC	*dom* qPCR
dom rev	AGCAGGAAGGGCACAGGAG	*dom* qPCR
domA fwd	AGAGTCCCAAGAAGCAGAAGA	*domA* qPCR
domA rev	ATCAGGCTCGGAGCACTAAAC	*domA* qPCR
domB fwd	GTCAACGGGGAAGGGAACAGA	*domB* qPCR
domB rev	CCTCGACGATCAAAGAGGCAT	*domB* qPCR
actin fwd	CAGAGCAAGCGTGGTATCCT	*actin* qPCR
actin rev	CTCATTGTAGAAGGTGTGGTGC	*actin* qPCR
Per CRS F	TGCCAGTGCCAGTGCGAGTTCG	CHIP *Per CRS* qPCR
Per CRS R	TGCCTGGTGGGCGGCTGG	CHIP *Per CRS* qPCR
Tim E-box1 F	ACGTTGTGATTACACGTGAGCCG	CHIP *Tim E-box* qPCR
Tim E-box1 R	TACACACACTGACCGAAACACCC	CHIP *Tim E-box* qPCR
FBgn0003638 F	ACTGCGTATTCAGGATACATGCC	CHIP intergenic region qPCR
FBgn0003638 R	TGTCCACTTTAATTGATTGCGTGG	CHIP intergenic region qPCR
